# Development of an efficient, non-viral transfection method for studying gene function and bone growth in human primary cranial suture mesenchymal cells reveals that the cells respond to BMP2 and BMP3

**DOI:** 10.1186/1472-6750-12-45

**Published:** 2012-08-03

**Authors:** Prem P Dwivedi, Peter J Anderson, Barry C Powell

**Affiliations:** 1 Craniofacial Research Group, Women’s and Children’s Health Research Institute, 72 King William Road, North Adelaide, South Australia 5006, Australia; 2 Discipline of Paediatrics, University of Adelaide, North Terrace, Adelaide, South Australia 5006, Australia; 3 Australian Craniofacial Unit, Women’s and Children’s Hospital, North Adelaide, South Australia 5006, Australia

**Keywords:** Transfection, Nucleofection, Skull, Bone, Primary cell culture, Mesenchymal, BMP2, luciferase

## Abstract

**Background:**

Achieving efficient introduction of plasmid DNA into primary cultures of mammalian cells is a common problem in biomedical research. Human primary cranial suture cells are derived from the connective mesenchymal tissue between the bone forming regions at the edges of the calvarial plates of the skull. Typically they are referred to as suture mesenchymal cells and are a heterogeneous population responsible for driving the rapid skull growth that occurs *in utero* and postnatally. To better understand the molecular mechanisms involved in skull growth, and in abnormal growth conditions, such as craniosynostosis, caused by premature bony fusion, it is essential to be able to easily introduce genes into primary bone forming cells to study their function.

**Results:**

A comparison of several lipid-based techniques with two electroporation-based techniques demonstrated that the electroporation method known as nucleofection produced the best transfection efficiency. The parameters of nucleofection, including cell number, amount of DNA and nucleofection program, were optimized for transfection efficiency and cell survival. Two different genes and two promoter reporter vectors were used to validate the nucleofection method and the responses of human primary suture mesenchymal cells by fluorescence microscopy, RT-PCR and the dual luciferase assay. Quantification of bone morphogenetic protein (BMP) signalling using luciferase reporters demonstrated robust responses of the cells to both osteogenic BMP2 and to the anti-osteogenic BMP3.

**Conclusions:**

A nucleofection protocol has been developed that provides a simple and efficient, non-viral alternative method for *in vitro* studies of gene and protein function in human skull growth. Human primary suture mesenchymal cells exhibit robust responses to BMP2 and BMP3, and thus nucleofection can be a valuable method for studying the potential competing action of these two bone growth factors in a model system of cranial bone growth.

## Background

The ability to transfect DNA into mammalian cells is vital in biomedical research, particularly in studies of mechanistic understanding and clinical application. In particular, functional analyses of proteins, their trafficking and localization, gene expression studies, tissue engineering and gene therapy frequently require introduction of plasmid DNA into mammalian cells. Some questions can be addressed using transformed cell lines but they are artificial models harbouring one or more oncogenic mutations that may have had widespread and poorly characterized effects on cellular biology, and reliance on their use alone can distort views of function and significance. In contrast, primary cultured cells are non-transformed and typically comprise a mixed population that is more representative of the cellular complexity of the tissue of origin. However, lipid-based methods commonly used to transfect transformed cells are generally ineffective with primary cells and achieving efficient transfection of primary cells is a common problem in biomedical research. The two major alternatives to lipid-based methods for introducing genes into cells are viral transduction and electroporation. Although viral transduction can be effective, construction and handling of viral vectors and the processes involved in transduction are time consuming and require specialist expertise and, in cases of clinical application, safety issues can become paramount [1-4]. Non-viral electroporation of genes into primary cells can provide a simple and efficient alternative but, typically, primary cells of different origins cannot necessarily be transfected using the same conditions, which then need to be optimized [5-10].

Growth of the skull vault occurs via a different process to that of long bone growth and less is known about the mechanisms involved. Whereas growth of long bones occurs via a two step process (known as endochondral ossification) in which chondrogenesis precedes osteogenesis [11-13], growth of the calvarial plates of the skull vault occurs via direct differentiation of mesenchymal osteoprogenitor cells into bone-forming osteoblasts. This occurs at the fibrous suture located between calvaria and is known as intramembranous ossification [13]. Premature bony fusion of cranial sutures results in craniosynostosis, a developmental disorder that affects one in 2500 live births [14], with dramatic consequences for affected children, including raised intracranial pressure, impaired vision and hearing, intellectual disability and psychological problems associated with different head shapes [15-18]. Human primary calvarial suture mesenchymal cells derived from the bone forming regions between the skull calvarial plates can be used to study the molecular mechanisms involved in skull growth and the premature bony fusion that characterizes craniosynostosis [19-21] in the pursuit of biologically-based, adjunctive therapies to minimize the need for recurrent surgical correction during early childhood development. However, lack of an efficient method for transfection limits progress towards this goal.

In this study we compared lipid-based and electroporation-based techniques to introduce genes into human primary calvarial suture mesenchymal cells and have shown that an optimized electroporation-based nucleofection technique provides the most effective and reliable transfection. We have conducted benchmark experiments to validate gene expression and study function using glypican 3 (*Gpc3*) a proteoglycan capable of regulating a variety of growth factors [22], and to measure cell responses to two important bone growth factors, bone morphogenetic protein 2 (BMP2) and BMP3, which have antagonistic roles in bone growth [23,24].

## Results

### Transfection of human primary calvarial suture mesenchymal cells using the Amaxa Nucleofector- II

**Figure 1:**
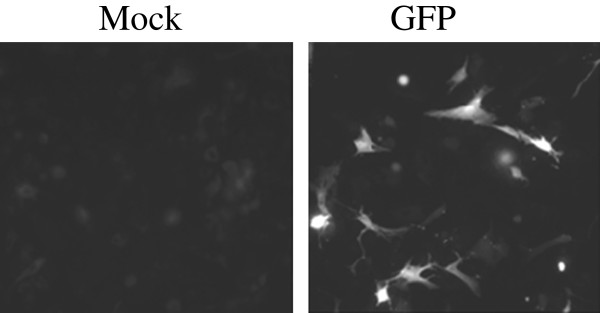
**Microscopic analysis of nucleofected*GFP* in human primary calvarial suture mesenchymal cells.** Human primary calvarial suture cells (1.0 x 10^6^) were transfected with 3 μg of pmaxGFP expression construct using Amaxa kit-L and the T030 program. The cells were imaged by light microscopy.

Initially, for transfection of human primary calvarial suture mesenchymal cells we tested several lipid-based, commercial transfection agents with a Green Fluorescent Protein (GFP)-containing expression construct, *pmaxGFP* (Table 1). None of the transfection agents, except endofectine and metafectine, showed any significant cell death as observed by light microscopy but the percentage of GFP-positive cells determined by flow cytometry indicated that these agents produced only 1-4 % transfection efficiency (Table 1). As a result of these very low transfection efficiencies, we tested electroporation-based methods using the Neon transfection system and the Amaxa Nucleofector II system. Using the Neon transfection system with human primary cranial suture mesenchymal cells, at best we achieved a transfection efficiency of 10 % (data not shown) with recommended amounts of plasmid DNA (500 ng *pmaxGFP)* and cells (100,000), and recommended optimization protocols that varied pulse voltage (850-1600 V), pulse width (10–40 ms) and pulse number (one to three pulses). In contrast, with the Amaxa Cell Line Optimization Nucleofector Kit transfection efficiencies of 35–56 % were achieved using program T030 and nucleofector solution-V (35 %) and nucleofector solution-L (56 %); however, cell survival was variable (Table 2). To optimize transfection efficiencies and cell survival we tested several other nucleofector kits (Basic Nucleofector Kit for Primary Mammalian Epithelial Cells and Cell Line Nucleofector Kit T for Bone Marrow) which have been used to transfect primary cells from other tissues. We never achieved transfection efficiencies above 10 % using these kits (data not shown). Therefore, we optimized plasmid concentration and cell number using the T030 program and nucleofector solution-L, which had initially produced the highest transfection efficiency in the human primary suture cells. We tested 3, 6 and 9 μg of *pmaxGFP* per transfection and found that increasing amounts of plasmid resulted in lower cell survival (Table 3). However, transfection efficiency was higher with more plasmid and the total number of transfected cells obtained with either 3 or 6 μg plasmid was similar. Next, we investigated transfection efficiency and cell survival using increasing cell numbers in the transfection mix (Table 4). Increasing cell numbers resulted in lower cell survival but higher transfection efficiency. The highest relative numbers of living transfected cells were achieved with inputs of 0.5 x 10^6^ and 1 x 10^6^ cells with 3 μg of plasmid. Figure 1 shows GFP fluorescence in living, transfected cells.

**Table 1:** Analysis of transfection efficiency of human primary calvarial suture mesenchymal cells by lipid-based transfection methods

**Transfection Method**	**% Cell Survival**	**% Transfection Efficiency**
Turbofect	ND	1.5±0.2
Lipofectamine 2000	ND	1.6±0.1
DOTAP	ND	1.4±0.1
X-Fect	ND	4.1±0.1
Endofectine	64.9±8.5	2.8±0.6
Metafectine	89.6±12.1	3.5±0.3

ND = not determined. Values ± SD represent the mean of 3 biological replicates.

**Table 2:** Effect of specific nucleofection programs on cell survival and transfection efficiency of human primary calvarial suture mesenchymal cells

		**V- Nucleofector buffer**		**L- Nucleofector buffer**	
**Nucleofection program**	**pmaxGFP (μg)**	**% Cell survival**	**% Transfection efficiency**	**% Cell survival**	**% Transfection efficiency**
No-Program control	3μg	99.2	0.2	98.8±0.7*	0.4±0.1*
T-030	No GFP control	26	0.3	37.0 ±7.0*	0.6±0.2*
T-030	3μg	14	35.5	19.0±2.0*	56.2±9.1*
A-020	3 μg	20	10.4	24	3.8
T-020	3 μg	14	22.7	17	31.6
X-001	3 μg	24	12.8	27	8.0
X-005	3 μg	24	16.5	20	13.7
L-029	3 μg	11	23.5	18	22.3
D-023	3 μg	15	27.2	20	14.8

Values ± SD with * represent the mean of 2 independent experiments, each with 2 from biological replicates. The other values are derived from one experiment with 2 biological replicates.

**Table 3:** Effect of increasing plasmid concentration on cell survival and transfection efficiency using kit-L and the T030 program

**Program**	**pmaxGFP (μg)**	**% Cell survival**	**% Transfection efficiency**
T-030	No GFP control	36.0 ±4.2*	0.7 ±0.2*
T-030	3 μg	19.0 ±1.2*	60.2 ± 6.7*
T-030	6 μg	13	90.8
T-030	9 μg	10	81.4

Values ± SD with * represent the mean of 3 independent experiments, each with 3 biological replicates. The other means are derived from one experiment with 3 biological replicates.

**Table 4:** Effect of increasing cells on cell survival and transfection efficiency using the cell line L kit and T030 program

**Program**	**Cells**	**pmaxGFP (μg)**	**% Cell survival**	**% Transfection efficiency**	**% Cell input, alive and transfected**
T-030	500,000	No GFP control	36.0 ±4.2*	0.4 ±0.2*	0.1
T-030	500,000	3 μg	19.0 ±0.8*	61.3 ±5.5*	11.6
T-030	1,000,000	3 μg	13.0 ±1.0^#^	88.5 ±2.3^#^	11.5
T-030	1,500,000	3 μg	11.0 ±1.2^#^	89.4 ±2.0^#^	9.8

Values ± SD with * represent the mean of 4 independent experiments, each with 4 biological replicates and values with ^#^ represent the mean of 2 independent experiments, each with 2 biological replicates.

### Validation of the nucleofection method demonstrates cellular responses to BMP2 and BMP3 in human primary calvarial suture mesenchymal cells

**Figure 2:**
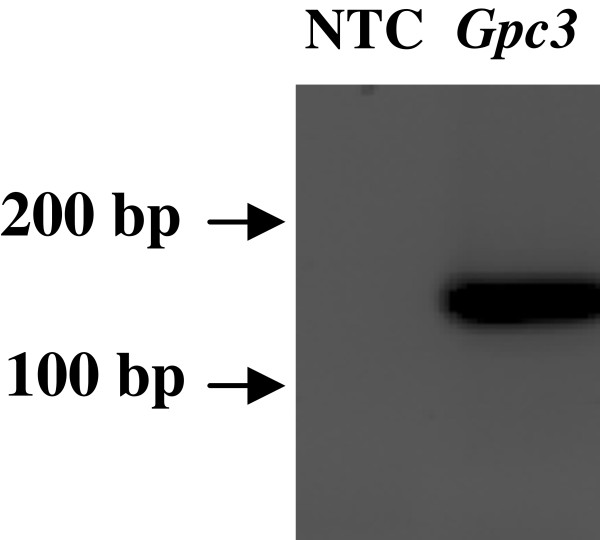
**Expression of nucleofected*Gpc3*.** Human primary calvarial suture cells (1.0 x 10^6^) were transfected with 3 μg of mouse *Gpc3* expression construct using Amaxa kit-L and the T030 program. The amplified PCR product (174 bp) shows the expression of mouse *Gpc3* while no template controls (NTC) shows no bands. The positions of 100 and 200 bp markers of a DNA ladder are indicated.

**Figure 3:**
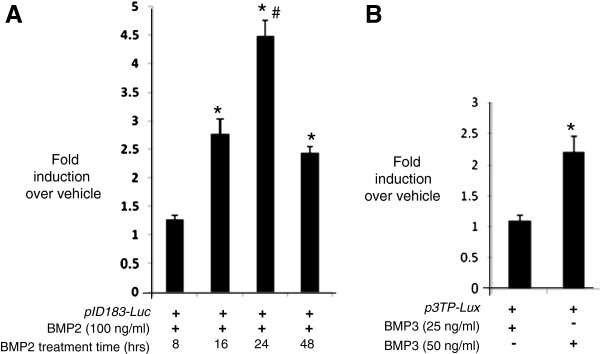
**Functional analysis of nucleofected BMP2 and BMP3-responsive promoter reporters.** Human primary calvarial suture cells (1.0 x 10^6^) were nucleofected with 3 μg of BMP2 responsive promoter construct (*pID183-luc*; 3A) or BMP3 responsive promoter construct (*p3TP-lux-Luc*; 3B) using Amaxa kit-L and the T030 program. The cells were also co-nucleofected with *pRL-TK-luc* construct as an internal control for transfection efficiency. Each construct was incubated with BMP2 or BMP3 or vehicle as indicated in the figures. The fold induction shown is the ratio of luciferase activity from BMP2-treated or BMP3-treated to vehicle treatment (ie no BMP) at the appropriate time point. In Figure 3A, * shows responses that are significantly different to vehicle, and to BMP2 treatment at 8 hrs, and # shows that 24 hrs BMP2 treatment is significantly different to all other time-points and treatments. In Figure 3B, * shows that the response to 50 ng/ml BMP3 is significantly different compared to vehicle and to the lower BMP3 dose. The data shows a representative result from three independent experiments, each performed with triplicate biological samples.

To demonstrate expression from other transfected plasmids and test functional responses in the cells, we first nucleofected a *Gpc3* expression vector into primary human calvarial suture mesenchymal cells using nucleofection. Expression of the nucleofected *Gpc3* was confirmed by RT-PCR (Figure 2). Next, cell function studies were conducted using the dual luciferase assay to test cell responses to two growth factor responsive luciferase reporter constructs, *pID183-Luc* and *p3TP-Lux,* responsive to BMP2 [25] and BMP3 [26] respectively. Twenty-four hrs post-transfection of *pID183-Luc*, BMP2 was added to transfected cells and luciferase activity of the BMP2 responsive construct was measured 8, 16, 24 or 48 hrs later. Maximal induction (4.5 fold) occurred 24 hrs post BMP2 treatment (Figure 3A). To investigate responses to BMP3, cells were transfected with *p3TP-Lux* and 24 hrs later BMP3 was added, then luciferase activity was assayed a further 24 hrs later. A dose of 50 ng/ml of BMP3 produced a 2 fold induction in luciferase activity (Figure 3B).

## Discussion

Inefficient introduction of plasmid DNA into primary cultures of mammalian cells is a common and frustrating problem in biomedical research that can restrict analyses of gene and protein function and limit progress in the understanding of human biology. Human primary cranial suture mesenchymal cells are an heterogeneous cell population derived from the bone forming regions between the calvarial plates of the skull and are responsible for driving the rapid skull growth that occurs *in utero* and postnatally. There are reports describing gene expression profiles of human cranial sutures in normal development and in abnormal medical conditions such as premature bony suture fusion in craniosynostosis, but limited information about how suture cell functions are regulated and the molecular consequences of genetic mutations on them [20,27,28]. This fundamental information can typically be derived by *in vitro* manipulation of gene and protein expression in cell-based assays, approaches that require efficient, simple and reliable cell transfection methods to enable general implementation. Lipid-based methods used to transfect plasmid DNA into mammalian cell lines are generally not effective for primary cells, as our assessment of several widely used lipid reagents demonstrated, and alternate methods based on electroporation have been developed. Since its first report 25 years ago by Chu et al. [29], electroporation of mammalian cells has been modified and developed as a commercially available technology for use in studying difficult to transfect cell types. However, primary cells of different origins respond differently, and methods need to be carefully optimized for each cell type. We evaluated two electroporation-based technologies, the Neon transfection system based on methodological advances developed by Kim et al. [30] and the Amaxa Nucleofector II system based on methodology developed by Amaxa GmbH in 1998. Using the Neon transfection system with human primary cranial suture mesenchymal cells, at best we achieved a transfection efficiency of 10 %, much lower than other studies using this system with human stem cells from bone marrow, adipose tissue and umbilical cord blood, where transfection efficiencies of 35-80 % have been reported [31-33]. In contrast, with the Amaxa Nucleofector II, transfection efficiencies of up to 90 % were attained, although cell survival varied with the transfection kits and programs tested. With optimization of transfection program, transfection solution, amount of plasmid DNA and cell numbers, a protocol was developed that resulted in an average of 60 % transfection efficiency with 20 % cell survival. This transfection efficiency is comparable to that achieved in other primary cells and in stem cells using the Amaxa Nucleofector or other electroporators [34-39]. Although mortality of the human primary suture mesenchymal cells is higher compared to other cell types, sufficient numbers of transfected cells can be obtained for quantitative-based assays of gene and protein function, as demonstrated using the dual luciferase assay.

BMP2 is the best known bone osteogenic factor and induces osteoblastic differentiation of multipotent mesenchymal cells [40,41]. In contrast, BMP3, a less well known BMP, antagonizes BMP2 action by competing for binding to a common effector, SMAD4 [42,43]. Loss of BMP3 results in excessive bone formation [42,43] and recent data show BMP3 suppresses osteoblastic differentiation and thereby is thought to limit the differentiation of osteoprogenitors [24]. Human primary suture cells have an osteoprogenitor-like phenotype and can be induced to differentiate with standard osteogenic supplements, ascorbic acid and β-glycerophosphate [19,21]. Cranial sutures express BMP2 [21,44] and BMP3 [45] and we used nucleofection of human primary suture cells to measure BMP2 and BMP3 signalling with luciferase reporter plasmids and demonstrate robust responses of the cells to both BMPs. It is possible that an interplay of these two BMPs may occur *in vivo* to influence in cranial bone growth, in which case that could provide an opportunity to manipulate and alter the outcome of abnormal bone growth in conditions such as craniosynostosis.

## Conclusions

A nucleofection protocol has been developed that provides a simple and efficient, non-viral alternative method for *in vitro* studies of gene and protein function in human skull growth. Human primary suture mesenchymal cells exhibit robust responses to BMP2 and BMP3, and thus nucleofection can be a valuable method for studying the potential competing action of these two bone growth factors in a model system of cranial bone growth.

## Methods

### Human primary calvarial suture mesenchymal cells

Human primary calvarial suture mesenchymal cells, a heterogeneous population derived from the connective mesenchymal tissue between the bone forming regions at the edges of the calvarial plates of the skull, were grown from human suture samples as described previously [21] and were obtained from patients undergoing surgical treatment for craniosynostosis at the Australian Craniofacial Unit of the Women’s and Children’s Hospital, Adelaide, South Australia. This work was approved by the Research Ethics Committee of the Women’s and Children’s Hospital (REC1033/10/2014) and was performed in compliance with the Helsinki Declaration for research involving human subjects. Written, informed consent was obtained from the child, parent or guardian according to the guidelines of the Research Ethics Committee of the Women’s and Children’s Hospital. Briefly, for experimental purposes, cells were grown in minimal medium (MM) consisting of high glucose Dulbecco’s modified essential medium (DMEM, Invitrogen Life Technologies, Gaithersburg, MD), supplemented with L-Glutamine (584 mg/l), 10 % foetal bovine serum (Invitrogen, Carlsbad, CA, USA), 1 % antibiotics (penicillin 100 IU/ml), streptomycin (100 μg/ml) and 1 % antibiotic:antimycotic (both purchased from Sigma-Aldrich, MI, USA) by incubating at 37C in an humidified incubator with 5 % CO_2_ in air. The cultures comprise cells from the suture mesenchyme and adjacent bone growth fronts that exhibit a pre-osteoblastic or osteoprogenitor phenotype.

### Transfection of human primary calvarial suture mesenchymal cells using various lipid-based methods

In preparation for transfection using various lipid based methods, cells were grown in 175 cm² flasks to 70-80 % confluency. On the day of transfection, cells were washed once with PBS and trypsinized by adding 2 ml of 1X trypsin (Sigma-Aldrich, St Louis, USA) and incubating the flask at 37C for 2 mins. The cells were stained with trypan blue and counted using a haematocytometer. Transfections using DOTAP (N-[1-(2,3-Dioleoyloxypropyl] N,N,N -trimethyl ammonium methyl sulfate; Roche Applied Biosciences, Indianapolis, USA), Lipofectamine 2000 (Life Technologies, Invitrogen, Australia), Turbofect (Fermentas Inc., Maryland, USA), X-Fect (Clontech Laboratories Inc., CA, USA), Endofectine (GeneCopoeia Inc., MD, USA) and Metafectine (Biontex Laboratories, GMbH, Germany) were carried out as described in manufacturer’s supplied protocols and as previously described [46-48]. Briefly for transfection, human primary suture cells (50,000 cells / well) were seeded in a 24-well tray and grown in 400 μl of minimal medium supplemented with 10 % fetal bovine serum. The cells were attached by incubating at 37C for 24 hrs. For each triplicate transfection, 750 ng *pmaxGFP* (green fluorescent protein) expression construct (3.49 kb: Amaxa Biosystems, now known as Lonza) was mixed with 30 μl of 20 mM Hepes buffer pH 7.4 in an eppendorf tube and, in another tube, the specific transfection agent was diluted with 30 μl of Hepes buffer to the recommended concentrations. The contents of these tubes were mixed and incubated at room temperature for 20 mins for DNA-lipid complex formation and 20 μl of this complex was aliquotted into three wells of a 24-well tissue culture tray. The next day the medium was removed and the cells washed once with 400 μl of phosphate-buffered saline (PBS) and replaced with 400 μl of medium, then incubated at 37C for another 24 hrs. Microscopy of the GFP-transfected cells was carried out using a Nikon Eclipse TE2000U inverted microscope attached with twin CCD cameras and 20x objective. Cells were then counted as indicated in the appropriate figure legends in an haematocytometer after trypan blue staining to calculate cell survival. Flow cytometric analysis of GFP-positive cells was conducted to determine transfection efficiency.

### Flow cytometry

The expression vector, *pmaxGFP*, was transfected or nucleofected into human primary calvarial suture mesenchymal cells. At 24 hrs post transfection cells were washed twice with PBS and dislodged from the tissue culture plates with 1X trypsin. Cells were then centrifuged at 1300 rpm for 10 mins and resuspended in 100 μl of PBS. 10 μl of these cells were used for trypan blue staining to calculate cell survival and 90 μl of the cells were further diluted to 200 μl in PBS for analysis of transfection efficiency by flow cytometry. GFP fluorescence was analyzed using a BD Bioscience FACS-AriaII. Dead cells were gated out using 7-Aminoactinomycin D (7-AAD) (5 μg/ml) staining. Data were analyzed using Flowjo Version 7 (Free Star Inc., USA).

### Nucleofection of human primary calvarial suture mesenchymal cells using Amaxa transfection kits

Nucleofection of human primary calvarial suture mesenchymal cells was carried out using the Amaxa-II Nucleofector method and available transfection kits. No kit was specifically available for the human primary calvarial suture mesenchymal cells under investigation in this study; therefore the Amaxa Cell Line Optimization Nucleofector Kit was tested for nucleofection of human primary suture cells. Briefly, in preparation for nucleofection, suture cells were dislodged from the tissue culture flask by trypsin and counted in a haematocytometer after trypan blue staining. All centrifugations were carried out with maximum g force of 150 g. For each nucleofection, suture cells were resuspended in 100 μl of V or L Nucleofector solution and 20 μl of the Amaxa Supplement in an eppendorf tube. The appropriate amount of *pmaxGFP* was also mixed as indicated in the respective tables and figure legends. Immediately, the mixture of suture cells, *pmaxGFP* and Nucleofector solution was transferred into an Amaxa cuvette and nucleofection conducted using the recommended program. The nucleofected cells were resuspended in 400 μl of minimal media and plated into a 24-well tissue culture tray. The cells were incubated overnight at 37C. The next day the medium was removed and the cells were washed three times with 400 μl of PBS and the medium replaced with serum-free DMEM and the cells incubated at 37C for a further 24 hrs. The following day, microscopy of the *GFP*-transfected cells was carried out and then cells were trypsinized and resuspended in 100 μl of PBS. Cells (10 μl) were counted in a haematocytometer after trypan blue staining to calculate cell survival. The remaining cells were analysed for GFP expression by flow cytometry analysis and transfection efficiency was determined.

### Construction of *Gpc3* expression vector

A mouse ORF cDNA (1.74 kb) for glypican 3 (*Gpc3*) and the gateway cloning vector, pcDNA-DEST40 (7.1 kb) were purchased (GeneCopoeia Inc., MD, USA). Gateway cloning was carried out essentially as described in the manufacturer’s protocol. The construct was verified using restriction digestion analysis and DNA sequence analysis (Applied Biosystems, CA, USA).

### Gene expression analysis of nucleofected *Gpc3* by RT-PCR

The *Gpc3* expression construct (8.84 kb) was nucleofected using optimized transfection conditions (Amaxa transfection kit L and program T030) for human primary calvarial suture mesenchymal cells. *Gpc3* nucleofected cells were grown in 24 well tissue culture tray for 24 hrs then washed three times with PBS and grown overnight. RNA was extracted using an RNAeasy mini kit (Qiagen, CA, USA) and cDNA synthesized from 200 ng RNA using a Superscript-III First Strand Synthesis kit (Invitrogen, CA, USA). Primers for *Gpc3* (forward 5’-ggttagccagatcattgacaaac-3’ and reverse 5’-cttcatcatcaccgcagtctc-3’) were synthesized (Geneworks, Adelaide, South Australia). The primers were specific for nucleofected mouse *Gpc3.* PCR reactions were carried out using 2 μl cDNA, 100X SYBR green (Abgene, Epson, UK), 1X Amplitaq PCR buffer, 2 mM MgCl_2_, 1 unit of Amplitaq (Applied Biosystems, Foster City, CA, USA), 0.4 mM dNTPs (Invitrogen, Carlsbad, CA, USA) and 5.625 μM primers in 25 μl. PCR was carried out using a Gene Amp PCR System 9700 (Applied Biosystems). An expected PCR product (174 bp) was resolved on a 1.5 % agarose gel. The PCR product was sequenced for confirmation (Institute of Medical and Veterinary Sciences, Adelaide, Australia).

### Functional analysis of BMP2 and BMP3 using promoter-reporter assays

For transient transfection analysis, human primary calvarial suture mesenchymal cells were grown in 175 cm² flasks to 70-80 % confluency. On the day of transfection cells were washed once with PBS and trypsinized by adding 2 ml of 1X trypsin and incubating the flask in a 37C incubator for 2 mins. The cells were stained with trypan blue and counted using a haematocytometer. Transfection was conducted using the Nucleofector kit L and program T030. In preparation for transfection, 1.5 x 10^6^ cells were resuspended in 100 μl of Nucleofector transfection solution with 3 μg of a BMP responsive promoter luciferase construct, *pID183-Luc* (5.78 kb) [25] or a TGF beta responsive promoter luciferase construct, *p3TP-Lux* (6.55 kb) [26] together with 500 ng of *pRLTK-Luc* construct (4.05 kb: Promega Corporation, USA) as a control to normalize transfection efficiency. The transfected cells were transferred to 3 ml of pre-warmed medium and 200 μl of cells were aliquotted to 96-well plates and incubated for 24 hrs in a 37C incubator with 5 % CO_2_. The following day cells were washed 3 times with PBS and then incubated with 200 μl of serum-free DMEM with antibiotic and BMP2 and BMP3 (R&D Systems, USA) as indicated in figure legends and cells were cultured for up to 48 hrs. Luciferase activity in cell lysates was determined using the Dual Luciferase Assay kit and a Luminometer as described previously [48,49].

### Statistical analysis

Results are presented as mean value of 2 to 4 biological replicates with standard deviation (SD) where appropriate. Specific assays were statistically analyzed using Analysis of Variance (ANOVA). The differences were considered significant when the P value was less than 0.05.

## Abbreviations

GFP, Green fluorescent protein; GPC3, Glypican 3; BMP, Bone morphogenetic protein; Luc, Luciferase.

## Competing interests

The authors declare no competing interests.

## Authors’ contributions

PPD, PJA and BCP designed the study. PJA collected human tissue samples and established the primary cultures. PPD performed the experiments. All authors contributed to the manuscript and have read and approved the final manuscript.

## Authors' information

PPD and BCP are molecular biologists. PJA is a craniofacial surgeon.
